# XPO1/CRM1-Selective Inhibitors of Nuclear Export (SINE) reduce tumor spreading and improve overall survival in preclinical models of prostate cancer (PCa)

**DOI:** 10.1186/1756-8722-7-46

**Published:** 2014-10-05

**Authors:** Giovanni Luca Gravina, Monica Tortoreto, Andrea Mancini, Alessandro Addis, Ernesto Di Cesare, Andrea Lenzi, Yosef Landesman, Dilara McCauley, Michael Kauffman, Sharon Shacham, Nadia Zaffaroni, Claudio Festuccia

**Affiliations:** Department of Biotechnological and Applied Clinical Sciences, Laboratory of Radiobiology, University of L’Aquila, L’Aquila, Italy; Department of Experimental Oncology and Molecular Medicine, Fondazione IRCCS Istituto Nazionale Tumori, Milano, Italy; Department of Biotechnological and Applied Clinical Sciences, Division of Radiotherapy, University of L’Aquila, L’Aquila, Italy; Department of Experimental Medicine, Pathophysiology Section, Sapienza University of Rome, Rome, Italy; Karyopharm Therapeutics, Natick, MA USA

**Keywords:** Prostate cancer, Bone metastases, Tumor suppressor protein, XPO1, CRM1, KPT-330, Selinexor

## Abstract

**Background:**

Exportin 1 (XPO1), also called chromosome region maintenance 1 (CRM1), is the sole exportin mediating transport of many multiple tumor suppressor proteins out of the nucleus.

**Aim and methods:**

To verify the hypothesis that XPO1 inhibition affects prostate cancer (PCa) metastatic potential, orally available, potent and selective, SINE compounds, Selinexor (KPT- 330) and KPT-251, were tested in preclinical models known to generate bone lesions and systemic tumor spread.

**Results:**

In vitro, Selinexor reduced both secretion of proteases and ability to migrate and invade of PCa cells. SINEs impaired secretion of pro-angiogenic and pro-osteolytic cytokines and reduced osteoclastogenesis in RAW264.7 cells. In the intra-prostatic growth model, Selinexor reduced DU145 tumor growth by 41% and 61% at the doses of 4 mg/Kg qd/5 days and 10 mg/Kg q2dx3 weeks, respectively, as well as the incidence of macroscopic visceral metastases. In a systemic metastasis model, following intracardiac injection of PCb2 cells, 80% (8/10) of controls, 10% (1/10) Selinexor- and 20% (2/10) KPT-251-treated animals developed radiographic evidence of lytic bone lesions. Similarly, after intra-tibial injection, the lytic areas were higher in controls than in Selinexor and KPT-251 groups. Analogously, the serum levels of osteoclast markers (mTRAP and type I collagen fragment, CTX), were significantly higher in controls than in Selinexor- and KPT-251-treated animals. Importantly, overall survival and disease-free survival were significantly higher in Selinexor- and KPT-251-treated animals when compared to controls.

**Conclusions:**

Selective blockade of XPO1-dependent nuclear export represents a completely novel approach for the treatment of advanced and metastatic PCa.

## Introduction

Prostate cancer (PCa) is the second leading type of cancer in men in european countries [1]. Deregulation of cell proliferation associated with bypassing of apoptosis, resistance to hormonal control and metastasis are all essential elements in the PCa progression. When confined to the prostate, PCa is effectively treated by surgery or radiotherapy. For patients with PCa that has escaped the prostate, the primary modality is to treat with androgen ablation therapy. Androgen ablation therapy provides variable periods of disease remission, but is not curative. Eventually, nearly all PCa becomes refractory to androgen deprivation and metastasizes to bone, leading to painfully debilitating fractures, spinal compression and rapid decline [2]. In addition, androgen deprivation resistant PCa bone metastases are usually resistant to standard therapies including radiation and chemotherapy. The major characteristic of PCa cancer bone metastases is that they typically produce osteoblastic or mixed osteoblastic/osteolytic bone lesions that are not as efficiently treated with the osteoclast inhibitors [3–6]. Treatments that target the bone microenvironment such as bisphosphonates including zolendronic acid [4] and the RANKL inhibitor, denosumab [5], which inhibits osteoclasts and associated osteolysis, have been effective in delaying the onset of skeletal related events (SREs) in some cancers, but mixed results in PCa. Novel therapeutic modalities that prevent and treat PCa associated SREs are needed.

Transport of macromolecules across the nuclear membrane is fundamental to the proper functioning of a living cell. For example, the ability to localize to the nucleus is essential for transcription factor activation, and spatial separation of proteins is commonly used as a mechanism for preventing spontaneous signal activation. XPO1/CRM1 (Exportin 1 or Chromosome Region Maintenance 1 protein) is one of seven mammalian export proteins that facilitates the transport of large macromolecules including RNAs and proteins across the nuclear membrane to the cytoplasm [7–10]. In addition to nuclear-cytosolic transport, XPO1 also plays a role in centrosome duplication and spindle assembly, especially in response to DNA damage [11]. Many cargo proteins that depend *exclusively* on XPO1 for nuclear export function as tumor suppressor proteins including p53, BRCA1, Survivin, NPM, APC, FOXO, and others, regulating cell growth and apoptosis [12–16]. An imbalance in the cytosolic level of these proteins has been observed in cancer cells, resulting in inactivation of tumor suppressor functions. Thus, the concept of inhibiting XPO1 has been explored as a potential therapeutic intervention in several tumors [17–22], but not in PCa. Because XPO1 inhibition impacts multiple anti-tumor and growth suppressive signaling pathways, it may impact PCa including those that have become androgen independent. A well-known natural product XPO1-specific inhibitor, Leptomycin, LMB, possesses strong anti-tumor activity in vitro, but phase I trials of LMB were discontinued because of its toxicity and lack of apparent efficacy in the tolerable dose range. Mutka et al. [23] noted that LMB has off-target effects against proteins other than XPO1, and that derivitization of LMB can ameliorate many of the side effects of LMB in mice, indicating that LMB’s off-target effects contribute to toxicities. The finding that inhibition of XPO1 itself was not the cause of LMB’s toxicity is promising in terms of the development of anticancer drugs targeting XPO1. In order to efficiently discover novel small-molecule, drug-like, selective inhibitors of nuclear export (SINE) that block XPO1-dependent nuclear export, we applied a virtual screening workflow based on a combination of protein modeling and simulations, physicochemical filters, and high-throughput molecular docking [23, 24]. SINEs are small molecule, drug-like compounds that form a slowly reversible covalent bond with Cys528 in the cargo-NES binding domain of XPO1 [17, 25]. These SINE compounds are active in a number of hematologic and solid tumor xenograft models. One of these compounds, Selinexor (KPT-330), which shows potent and selective inhibition of XPO1 and has good oral bioavailability in animals [25–27], has entered phase 1 studies in patients with advanced solid tumors (clinicaltrials.gov:NCT01607905). The objective of our study was to test the hypothesis that XPO1 inhibition affects the metastatic potential of PCa cells using one model of intraprostatic tumor growth and two models of bone metastasis.

## Materials and methods

### Materials

All the materials for tissue culture were purchased from Hyclone (Cramlington, NE, USA). Plasticware was obtained from Nunc (Roskilde, Denmark). P27 (C19, sc-528), CRM1 (H300, sc-5595), FAK (C-903, sc-932), β-actin (sc-130065) and Lamin B (C-20, sc-6216) were purchased from SantaCruz Botechnology Inc (Heidelberg, Germany). ELAV1/HuR1 antibody was purchased from Aviva systems biology Corporation (San Diego, CA, USA). KPT-251 and Selinexor (KPT-330) are two structurally similar, selective XPO1 inhibitors with distinct pharmacokinetic (PK) properties were provided by Karyopharm Therapeutics Inc. (Natick, MA) [21, 23]. Alendronate (Ale) and poly (2-hydroxyethyl methacrylate) (poly-HEMA) were purchased from Sigma-Aldrich (St Louis, MA).

### Cell lines

Androgen independent PC3 and DU145 cell lines were obtained from ATCC (LGC standards, Teddington, UK). Bioware Ultra Cell line, PC3M-pro4-luc cells were kindly provided from G. van der Pluijm (Uro-Oncology Research Laboratory, Leiden, The Netherlands PC-3 M-Pro4/luc + cells were maintained in Dulbecco modified Eagle medium (GibcoBRL, Breda, The Netherlands) containing 4.5 g glucose/L supplemented with 10% FCII, 100 U/ml penicillin, 50 μg/ml streptomycin, and 800 μg/ml geneticin/G418 (Invitrogen, Breda, The Netherlands) as described previously [28]. PCb2 [29, 30] cells were derived from PC3 cells after two passages in mice (tibiae) and showed a higher bone metastatic capacity when compared to parental cells or PC3-M cell derivatives [31, 32]. The mouse monocytic cell line RAW 264.7 [33] was kindly provided from Prof E. Tolosano, University of Torino, Torino, Italy. RAW264.7 cells were cultured in DMEM (Dulbecco’s modified Eagle’s medium) containing 4.5 g/l glucose and supplemented with 10% (v/v) heat inactivated FBS, 0.5 units/ml penicillin and 50 μg/ml streptomycin.

### Suspension culture and anoikis protocol

Regular cell culture dishes were coated with a film of poly-HEMA following the protocol as previously reported [34]. Briefly, a solution of 120 mg/ml of polyHEMA in 95% ethanol was mixed overnight, centrifuged at 800 g to remove undissolved particles and diluted 1:10 in 95% ethanol. The resulting solution was then placed in the culture dishes. The dishes were left to dry at room temperature overnight. Before use, the coated dishes were washed twice with phosphate buffered saline (PBS). Near-confluent PC3, PC2b and DU145 cells on regular plastic tissue culture dishes were trypsinized to produce a single cell suspension. Cells were suspended in growth medium in the presence of 0.5% methylcellulose (to avoid potential survival effects caused by cell clumping) in suspension at a density of 500,000 cells/ml. Approximately 2.5 × 10^5^ cells were placed in each well of a six well dish pre-coated with poly-HEMA to prevent cell attachment. Cell suspension was then removed from the plate, and wells were washed with 1× PBS to collect any residual cells. Cells pellets were obtained through centrifugation then washed twice with 1× PBS and processed for specific assays. At the indicated times, cells were collected, washed with PBS and resuspended by trypsinization. Viable cells were counted using the NucleoCounterTM NC-100 (automated cell counter systems, Chemotec, Cydevang, DK). Apoptosis was evaluated by using Tali® Apoptosis Kit - Annexin V Alexa Fluor® 488 & Propidium Iodide-based, (Life Technologies Italia, Monza, Italy). Stained cells were then measured on a Tali® Image-Based Cytometer. Apoptosis was further confirmed by cytofluorimetric analysis following the instructions of producer.

### Migration and invasion assay

Cell migration and invasion assays were performed after appropriate treatments using Boyden chambers containing PVPF 8 μm polycarbonate filters (Nucleopore, Concorezzo, Milan, Italy) coated on one side with 10 μg/ml type I collagen or 25 μg/ml of matrigel (Becton Dickinson Italia, Milan, Italy), respectively. Tests have been performed as previously described [33].

### Protease expression

The expression and activation of gelatinase A (pro-MMP-2) and gelatinase B (pro-MMP-9) were analyzed by zymography performed using SDS-polyacrylamide gel copolymerized with 0.1 mg/ml gelatin [35, 36]. For plasminogen activator analysis, gels were performed copolymerizing SDS-polyacrylamide with 0.1 mg/ml of lactose-free casein and 15 mg/ml of human plasminogen B as previously described [34]. Conditioned media from PCa cells treated or not with KPT330 (7 mM) were obtained from cultures grown onto 24-well plates. Following incubation, culture supernatants were collected, centrifuged at top speed in a Eppendorf Microcentrifuge (Hamburg, Germany) for 5 min to remove cell debris and stored at -80°C until assayed. Corresponding monolayers were trypsinized and the cells counted in a Neubauer chamber (Hausser, Blu Bell, PA, USA) to normalized the gelatinase activity of the conditioned media.

### Primary cultures of mouse calvarial osteoblasts

Osteoblast cultures were derived from calvaria of 6- to 9-day-old CD1 mice. The bone samples were sequentially digested with 1 mg/ml Clostridium histiolyticum type IV collagenase (Sigma) and 0.025% trypsin in Hank’s buffered solution. Cells from second and third digestions were grown in DMEM with antibiotics and 10% FBS. At confluence, cells were trypsinized, counted and plated in appropriate vessels for experiments. The osteoblast phenotype was evaluated by cytochemical analyses of ALP activity using reagents and protocols from the Sigma-Aldrich kit 104-LS. Total numbers of cells and numbers of ALP-positive cells will be mean counts of three microscopic fields from at least three cultures of (magnification 20×). Moreover densitometry of the wells was performed. Conditioned media were harvested from sub-confluent cultures treated with serum free fresh medium for 24 hr.

### Osteoclastogenesis assay

RAW264.7 cells were incubated with PC3 and PCb2 conditioned medium collected from untreated or treated with KPT-330 (7 μM) cells in the presence or not of 50 ng/ml RANKL. Media was replaced every 48 h and after 5 d, cells were stained for tartrate resistant acid phosphatase (TRAP) using Sigma TRAP staining kit (Sigma Diagnosis, St. Louis, MO). The number of TRAP positive cells (a measure of osteoclast activity) was determined using a light microscope. Cells were processed further as per the protocol and differentiated osteoclasts (with four or more nuclei) were counted. RAW264.7 cells supplemented with 5 ng/ml of RANKL only served as negative control. In order to verify whether KPT compounds possessed direct effects on RAW264.7 cell differentiation, we supplemented KPT-330 to RAW264.7 cells treated with RANKL (50 ng/ml) and analyzed for osteoclast activity and differentiation.

### In vitro cytokine analyses

DU145 and PC3 cells were seeded onto 24-well plates at a cell count of 2 × 104 cells/well in a regular culture medium and incubated at 37°C for 48 h in the absence and the presence of KPT-330 and KPT- 251 at concentrations as indicated. The conditioned medium was collected and assayed with a sandwich ELISA for TGF-β1 (RayBiotech, Inc., Norcross GA), IL-8 (Biorbyt ltd, Cambridge, UK), IL6 (Biorbyt ltd), VEGF (Biorbyt ltd) and RANKL (Enzo Life Sciences, Inc. Italian distributor Vinci-Biochem Florence) according to the manufacturer’s protocol.

### Western blot

Cytoplasmic and nuclear protein extracts were obtained by using the Nuclear/Cytosol Fractionation Kit, Catalog #K266-25, from Biovision Inc. (Milpitas, CA, USA). Cell extracts and Conditioned media from treated and untreated cells were electrophoresed under reducing conditions and transferred to nitrocellulose filter (Schleicher and Schuell GmbH, Dassel, Germany). Non specific binding sites were blocked for 1 h in 5% non-fat dried milk in a Tris buffer containg 20 mM tris and 137 mM NaCl (pH 7.6). Blots were incubated with 1 μg/ml of primary antibody diluited in blocking solution for 1 h at room temperature, washed and then incubated for 1 h in secondary antibody diluted 1:3000 in blocking solution. Following a further wash, reactive bands were visualized by a chemiluminescent detection kit (Supersignal, Perbio Science, Tattenhall, UK) b y using Bio-Rad gel Doc™ (Bio-Rad Laboratories S.r.l., Miulan, Italy).

### In vivo treatments

#### Orthotopic model

Eight week old male SCID mice (Charles River, Calco, Como) were used. Experimental protocols were approved by the Ethics Committee for Animal Experimentation of the Fondazione IRCCS Istituto Nazionale dei Tumori (Milan, Italy). Mice were housed in a pathogen-free facility with food and water available ad libitum. Animal were anesthetized using a solution of Ketamine (100 mg/kg) and Xylazine (20 mg/kg), delivered i.p. in a volume of 10 ml/kg of body weight. All surgical procedures were carried out in sterile condition under a 10 × microscope (SMZ800 Nikon). Briefly, abdominal wall muscles were incised, and the bladder and seminal vesicles were delivered through the incision to expose the prostate. One million DU-145 cells in 50 μl of RPMI were injected in the right posterior prostatic lobe using a 1 ml syringe with a 27 Gauge needle [32, 37] The incision was closed with Polyglactine 910 (Vicryl 5–0) and surgical staples. Experiments were started when tumors were palpable. A tumor was defined as “palpable” when it is detected by palpation and confirmed upon histological analysis (performed in a couple of mice that were sacrificed to this purpose). At 35 days after cells injection, when intraprostatic tumors were palpable, mice were randomized into experimental groups (8–9 mice/group) to receive either vehicle or KPT-330. At this time, tumor weight was about 100 mg. Such a tumor weigh is a common starting point for drug activity evaluation in human tumor xenografts subcutaneously or orthotopically implanted. The drug was dissolved according to Karyopharm’s instructions and delivered by oral gavage 2 times a week for 7 times at a dose of 10 mg/kg or every day for 5 days at dose of 4 mg/kg. All mice were monitored biweekly for tumor onset. At the end of the experiment (65 days after cell injection), all the mice were anesthetized and sacrificed for the evaluation of drug efficacy. At necroscopy, intra-prostate tumors were collected and weighted. Peritoneal surface and diaphragm were inspected macroscopically for the presence of metastatic nodes and tumor specimens were fixed in 10% buffered formalin for subsequent histological analysis.

#### Intracardiac tumor model

**Figure 1:**
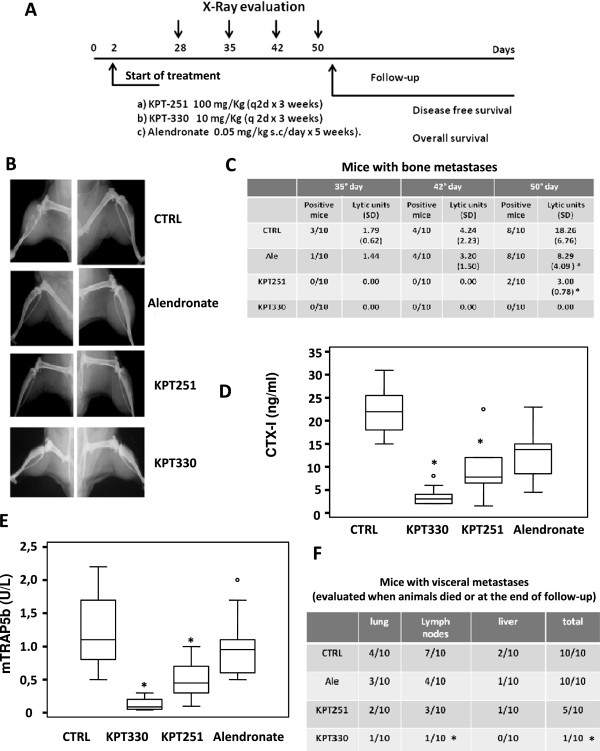
**Pharmacological treatment with XPO1 inhibitors reduces incidence and increases survival in a model of prostate cancer derived bone metastasis. (A)** In vivo schematic representation of the treatments in bone metastatic PCb2 model. **(B)** Representative X-ray pictures of untreated (vehicle), alendronate or selinexor ( KPT-330) and KPT-251 treated mice collected at 50^th^ day after intracardiac cell injection and showing strong osteolysis in untreated animals, moderate osteolysis in alendronate and KPT-251 treated animals and no lesions in selinexor treated animals. **(C)** table summarizing incidence and osteolysis (Lytic units) as measure of tumor burden at 35, 42 and 50 days after tumor inoculation and treatments. **(D)** serum CTX-I and **(E)** mTRAP levels measured in control animals and upon treatments. Both panels show the levels of CTX-I and TRAP measured before the death of the animals or in survived animal at the end of follow-up (170 days) **(F)** Visceral metastases incidence upon treatments in intact nude mice intracardiacally injected with PCb2cells. *P at least <0.05.

Heart injection of PCb2 cells was performed as previously described [29, 30]. Briefly, a 27 gauge needle on a tuberculin syringe containing 1 × 10^5^ tumor cells in 0.1 ml of PBS was inserted in the second left intercostal space of male CD1 nu/nu mice. Two days after treatments with XPO1 inhibitors, Selinexor [10 mg/Kg (q 2d × 3 weeks) or KPT-251 [100 mg/Kg (q 2d × 3 weeks) p.o.] were started (Figure 1A). We used alendronate (0.05 mg/kg s.c.) as internal positive control since in our previous experience demonstrated to be able to reduce osteolysis [38]. All treatments were started two days after cell injection in order to study how XPO1 inhibitors affect the growth of a limited number of tumor cells within bone marrow. This experimental model mimics the clinical condition of patients without clinical evidence of bone lesions but at high risk of bone metastasis development (e.g., with castration resistant PCa). The development of metastases was monitored by radiography using a Faxitron cabinet x-ray system (Faxitron x-ray corp., Wheeling, IL, USA ). Radiographic analyses were performed at days 28, 35, 42 and 50 after cell injection. No Faxitron analysis was performed after the 50th day since after this time the estimated risk of anesthesia-related mortality of mice was significantly increased. However, in order to determine both cumulative incidence of bone metastases and disease free survival (DSF), X-rays were also repeated at the death of each animal or in the survived animal at the end of follow-up, that we have defined to be 170 days, when animals were sacrificed. We previously demonstrated [29] that intra-ventricular injection indicate a low probability of developing forelimb tumors and high probability of developing hind limb tumors. For this reason we have focused on analyzing the metastases in the hind femurs and tibias. Burden of osteolytic lesions was evaluated by digital examination of radiography (ImageJ, a public domain software by Wayne Rasband, NIH, USA). Animals were sacrificed by carbon dioxide inhalation 170 days after heart injections, or earlier if there were early signs of serious distress. All animals were subjected to an accurate post mortem examination and samples of various organs were processed for routine histological analyses.

#### Intratibial tumor model

Intratibial tumor injection was performed as previously described [31, 32, 39]. Briefly, luciferase transfected PC3Luc cells were injected at 2 × 10^5^ cells/10 μl of serum-free medium into the proximal tibiae of 4-week old male CD1 nu/nu mice. The development of metastases was monitored by using a Faxitron cabinet x-ray system and tumor burden evaluated by bioluminescence analyses (see below).

#### Treatments of bone metastases models

Before drug administration, animals were divided into four groups (n = 10) and treated by oral gavage: Group 1 receiving vehicle control (Pluronic F-68/PVP-K29/32), Group 2 receiving KPT-251 [100 mg/Kg (q 2d × 3 weeks) p.o.]; Group 3 receiving KPT-330 [10 mg/Kg (q 2d × 3 weeks) p.o.]: group 4: mice (10 animals) receiving alendronate (0.05 mg/kg s.c.).

#### Assessment of treatment response in bone metastatic models

Tumor and bone treatment response was determined using X-rays as well as histomorphometric analyses of H&E stained sections. Skeletal lesions were calculated as described by Yang et al. [40], where 0 = no X-ray detectable lesions, 1 = minor lesions, 2 = small lesions, 3 = significant lesions with minor break of margins, or 4 = significant lesions with major break of margins.

#### Bioluminescence analyses

For luminescence imaging, mice received 150 mg firefly luciferase (Synchem Ug and Co.KG, Felsberg-Altenburg, Germany) per kg body weight given intraperitoneally. Following anesthesia with ketamine/xylazine mixture mice were placed into a Hamamatsu imaging station (Hamamatsu photonics, Italian distributor, Rome Italy). Bioluminescence generated by the luciferin/luciferase reaction was used for quantification using a dedicated Living Image software on a red (high intensity/cell number) to blue (low intensity/cell number) visual scale. A digital grayscale animal image was acquired followed by acquisition and overlay of a pseudo-color image representing the spatial distribution of detected photon counts emerging from active luciferase within the animal. Signal intensity was quantified as the sum of all detected photons within the region of interest during a 1-minute luminescent integration time. Tumor incidence was scored on a dichotomous scale as being either positive or negative if animals had at least one lesion detected in either the humeri or tibia/femur region.

#### Manipulation of bone metastatic tissues

For histologic examination, tibiae and femurs were dissected, cleaned from soft tissues, and fixed in 4% formaldehyde in 0.1 mol/L phosphate buffer (pH 7.2). Samples were then decalcified in EDTA and embedded in paraffin. Sections were cut and stained with H&E to evaluate the displacement of bone marrow by tumor cells as well as the tumor diameter.

#### Tartrate-resistant acid phosphatase staining

For the detection of osteoclasts, TRAP staining was done using Sigma Diagnosis Acid Phosphatase Kit. The number of TRAP-positive osteoclasts at the tumor-bone interface was counted under a microscope within 5 individual microscopic 200× fields.

#### Serum levels of CTX-I and TRAP-5b

Blood samples were collected from the tail of animals at defined times. For the intraventricular model we collected blood samples every two weeks or when the general conditions of the animals were indicative of impending death. For the intra-tibial model blood samples were collected at 10, 15, 28 and 35 days. Measurement of serum Mouse cross linked C-telopeptide of type I collagen (CTX-I, USCN Life Science Inc., Houston, TX) and mouse TRAP-5b (mTRAP, MyBioSource, Inc., San Diego, CA) were performed according to the manufacturer’s protocol. Blood without anticoagulant agents was collected, allowed to clot and then centrifuged at 2,000 × g for 10 min to obtain serum, which was stored at -20°C until further assay.

#### Statistical analysis

Continuous variables were summarized as mean and S.D. or 95% CI for the mean. Statistical comparisons between controls and treated groups were established by carrying out the ANOVA test or by Student’s t test for unpaired data (for two comparisons). Dichotomous variables were summarized by absolute and/or relative frequencies. For dichotomous variables, statistical comparisons between control and treated groups were established by carrying out the exact Fisher’s test. For multiple comparisons, the level of significance was corrected by multiplying the P value by the number of comparisons performed (n) according to Bonferroni correction. Overall and Disease-Free survival were analyzed by Kaplan–Meier curves and Gehan's generalized Wilcoxon test. When more than two survival curves were compared, the Logrank test for trend was used. This tests the probability that there is a trend in survival scores across the groups. All tests were two-sided and were determined by Monte Carlo significance. P values <0.05 were considered statistically significant. SPSS (statistical analysis software package, IBM Corp., Armonk, NY, USA) version 10.0 and StatDirect (version. 2.3.3., StatDirect Ltd, Altrincham, Manchester, UK) were used for statistical analysis and graphic presentation.

## Results

### Selinexor (KPT-330) and KPT-251 reduce incidence of bone metastasis and increases survival of mice with bone metastases after intraventricular injection of tumor cells

**Figure 2:**
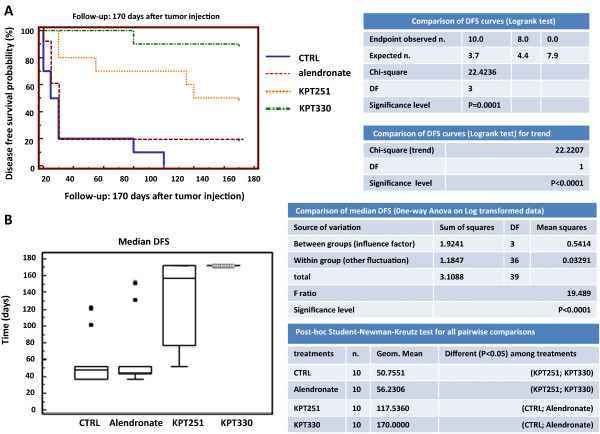
**Intraventricular cell injection. (A)** Analysis of Disease Free Survival by Kaplan-Meier curves and logrank test upon KPT-251, KPT-330 and alendronate treatments. **(B)** Median Disease Free Survival, one-way Anova on log-transformed data and post-hoc Student-Newman-Keults test for all pair wise comparisons.

**Figure 3:**
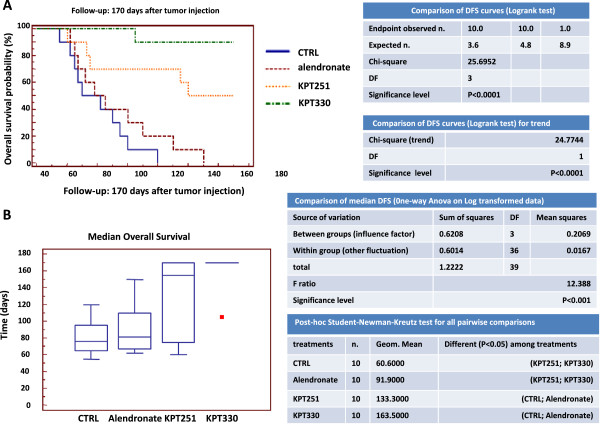
**Intraventricular cell injection. (A)** Analysis of Overall Survival by Kaplan-Meier curves and logrank test upon KPT-251, selinexor (KPT-330) and alendronate treatments. **(B)** Median Overall Survival, one-way Anova on log-transformed data and post-hoc Student-Newman-Keults test for all pair wise comparisons.

PC2b cells, an aggressive variant of PC3 cells, were injected in CD1 nu/nu male mice treated accordingly to the schema in Figure 1A. Forelimb osteolytic bone lesions (Figure 1B) appeared in control mice at day 35 post-inoculation in 3 out of 10 (30%) mice and progressively increased to 80% (8/10) at the 50^th^ day (Figure 1C). In contrast, no osteolytic lesions were detected until day 50 in KPT-251 treated animals (2/10, Figure 1C) although the overall incidence at the end of experiment (day 170) was 50% (5/10). Interestingly, 0/10 animals showed bone metastases after Selinexor treatment in any X-ray analysis (P < 0.0001 vs vehicle-treated animals) and no animals had bone lesions at the end of experiment (day 170), whereas 1/10 of animals died with visceral metastases after 100 days (P < 0.05 vs vehicle-treated animals). Bone lysis measured as lytic units was significantly higher in CTRL with respect to KPT-251 (p < 0.05) (Figure 1C) whereas no osteolysis was observed after Selinexor treatment. In agreement with previous data literature, alendronate treatment did not alter the incidence of bone lesions, but tumor-induced osteolysis was reduced [41–43]. Serum levels of CTX-I (Figure 1D) and mTRAP5b (Figure 1E), two specific markers of bone reabsorption, were significantly lower in the KPT-251 in treated animals with respect to CTRL (p < 0.005) and this paralleled with the reduced bone lysis found in these animals. In Selinexor treated animals, the levels of these markers were similar to those observed in animals which underwent empty injections without tumor cells and significantly lower than levels in CTRL and KPT-251 treated groups (p < 0.001). Not statistical significance were observed between alendronate and CTRL groups, whereas both KPT251 and KPT-330 treated animals showed significantly lower levels of CTX and mTRAP to those measured in alendronate-treated animals (p < 0.005). Post-mortem necroscopy documented lower incidence of lymph node, lung and liver metastases upon KPT 251- and Selinexor- treatments (Figure 1F). However, the one animal that died in the KPT-330 treated group had a large lung metastasis and involvement of loco-regional lymph nodes.The Kaplan-Meier curves showed that KPT-251 and Selinexor both improved DFS (Figure 2A-B) and OS (Figure 3A-B) of mice with bone lesions obtained by intracardiac injection compared with CTRL and mice treated with alendronate. However. no statistically significant difference was found between KPT-251and KPT-330 treated animals in terms of DFS and OS.

### Anti-metastatic effects is affected by number of tumor cells within bone marrow

**Figure 4:**
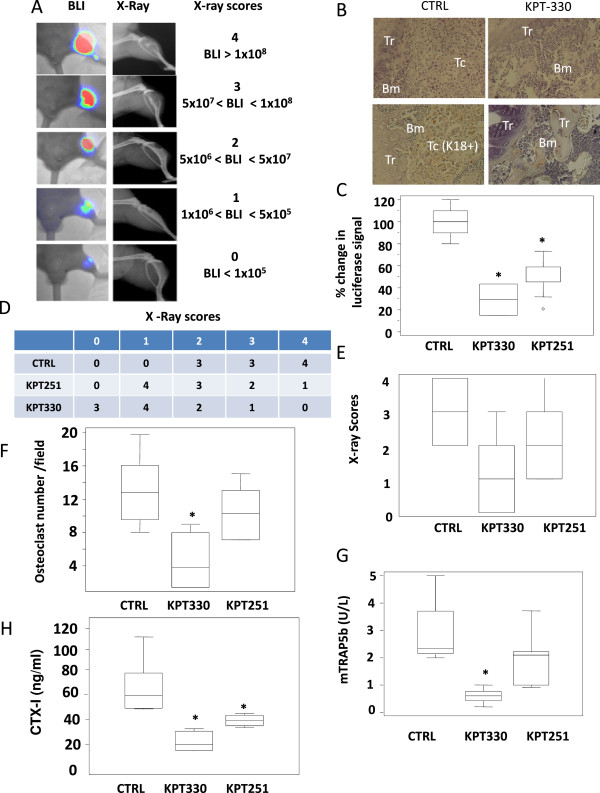
**Intratibial injection of PC3 cells.** Treatments with KPT-251 and selinexor (KPT-330) started two day after cell inoculation. **(A)**. Representative X-ray and bioluminescence pictures of untreated (vehicle) or KPT-251 and selinexor treated mice collected at 50^th^ day after intratibial cell injection. **(B)** Tibiae injected with PC3M-pro4 luc cells from mice of controls (CTRL) and treated with KPT330 and visualized at low (100×) and high (400×) magnification. This panel presents in the upper side a hematoxylin/eosin staining showing a strong tumor cell colonization and very low/absent tumor cell colonization which is detected by immune-hystochemical evaluation of cytocheratin 18 (K18). In the CTRLs tumor cell form a compact structure with reduced bone stroma cell presence. Tumor cells colonize also the cortical bone after having supporting osteolysis. No PC3 tumor cell colonization associated with any macroscopic variations in the bone stroma cell population was evident after KPT330 treatments. Tr (bone trabecula), Bm (bone marrow), Tc (tumor cells). **(C)** graph showing the percentage of change in luciferase signal. **(D)** Number of radio logically evident intratibial bone lesions having scores ≤ 2 and ≥ 3 according to Yang et al. procedure [40]. **(E)** X ray scores. **(F)** osteoclast number evaluated in the bone metastatic lesions. Osteoclasts are visible as histochemical purple staining for TRAcP activity as a marker of osteoclasts. Data are expressed as number of osteoclasts/mm at the tumor–bone interface. Number of osteoclasts was proportional to the lysis score but no statistical significance was observed. NE = not evaluable. The NE cases were excluded by analyses. **(G)** mTRAP and **(H)** CTX-I levels in controls and upon 35 days of KPT-330 and KPT-251 treatments in intratibial injected mice with PC3 cells.

In order to discern if the number of tumor cells within bone marrow (i.e., the intraosseous tumor burden) was a variable related to the effectiveness of KPT-251 and Selinexor in terms of reduced incidence of bone metastases, Luciferase-transfected PC3 cells were directly injected into tibia of nude mice. This model allowed us to inject a substantially higher number of tumor cells within bone marrow in comparison with the intracardiac tumor model. Consistent with our previous data [29, 30], 3 to 5 days after intra-tibial tumor cell injection, bioluminescence imaging revealed a significant intraosseous presence of PC3-Luc cells in all injected mice. BLI analyses were confirmed to be osteolytic lesions whose intensity was correlated with the size of bone metastasis (Figure 4A). Tumor cells grow in the bone marrow in close proximity with the trabeculae of the proximal epiphysis and very few cytokeratin positive cells are found in the bone marrow of selinexor treated mice (Figure 4B). All CTRLs were shown to have radiologically evident lesions and tumor cell component was prevalent in CTRL animals in comparison with Selinexor treated animals. Selinexor reduced the growth of tumor cells in the bone marrow as indicated to the changes in luciferase signal (Figure 4C). Also KPT-251 treatment reduced tumor growth (lower BLI intensities, Figure 4C) and lower incidence of radiographic lesions with high scores (Figure 4D,E) in comparison with CTRL. Osteolysis was also reduced as indicated by reduction in X-ray scores (Figure 4D,E) and osteoclast number (Figure 4F). Osteoclasts number was calculated by the direct count of purple staining TRAP positive cells (osteoclasts) at the interface tumor/bone. We considered for this analyses tibiae having a score higher of 1 since was difficult to found a sufficient area in which tumor cells were in contact to bone trabeculae. Therefore we analyzed three different sections of 10/10 tibiae of control mice, 7/10 tibiae of mice treated with KPT251 and 5/10 tibiae of mice treated with selinexor. We found that osteoclast number was, however, proportional to extension of lytic lesions and very similar in animals having similar lesions also if different treatments were administered. Nevertheless, osteoclast number was higher in control animals (11.2 +/- 5.4 cells/filed) when compared to selinexor (5.3 +/- 3.6) or KPT251 (8.4 +/- 2.6) treated animals also if statistical significance was observed only in selinexor treated mice (p = 0.033). Reductions in the serum levels of CTX-I and TRAP5b in treated versus CTRL animals (Figure 4G,H) confirmed once again the ability of Selinexor and KPT-251 in reducing osteoclast activity.

### Selinexor reduces tumor spread of PCa cells orthotopically injected into prostate gland

**Figure 5:**
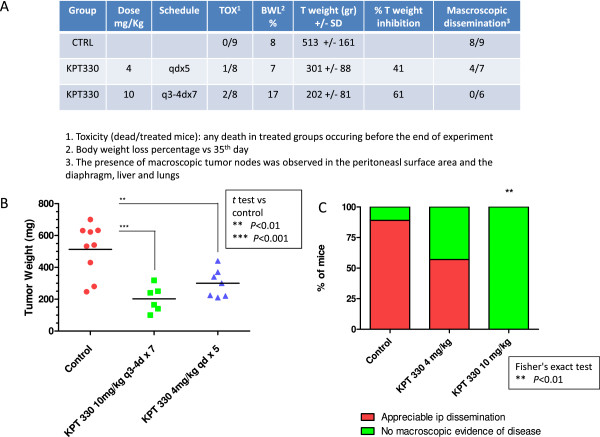
**Orthotopically implanted DU-145 human prostate carcinoma cells.** At day 65 (i.e., 26 days after the last treatment with selinexor (KPT-330) at the dose of 4 mg/kg, and 7 days after last treatment with selinexor at the dose of 10 mg/kg) mice were sacrificed for evaluating antitumor activity of the drug. **(A)** table summarized the pharmacological effects of selinexor including Toxicity, Body weight Loss (BWL), tumor mass weight, tumor mass reduction and macroscopic dissemination in the peritoneal surface and diaphragm. **(B)** single tumor mass weight in different treatment groups. **(C)** percentage of mice with macroscopically evident dissemination.

To further confirm that XPO1 inhibition may represent a therapeutic strategy in PCa, we used this orthotopic model to determine if SINE compounds could control local disease. In particular, we used the DU145 cells line which have not elevated capacity to metastasize, also if causes wide visceral diffusion when injected into prostate gland [44]. Since our experimental evidence in bone metastatic models suggest that Selinexor possesses higher antitumor and anti-bone metastatic activity when compared with KPT-251, Selinexor was used for the prostatic orthotopic experiments. SINE treatment was started when prostate (tumors) were palpable.At day 65 (i.e., 26 days after the last treatment with Selinexor 4 mg/kg, and 7 days after last treatment with Selinexor 10 mg/kg), mice were sacrificed for evaluation of antitumor activity. Selinexor induced statistically significant tumor weight inhibition compared to CTRL mice (41%, p < 0.01, for 4 mg/kg, and 61%, p < 0.01 for 10 mg/k, respectively, compared with CTRL). In addition, while a macroscopic tumor dissemination in the peritoneal surface and diaphragm was recorded in 8 out of 9 control mice, the presence of metastatic nodes was appreciable in only 4 out of 7 mice treated with the daily schedule (4 mg/kg × 5 consecutive days) of KPT and in no animal treated with the biweekly schedule (10 mg/kg × 7 administrations). Toxic effects in this model were in 1 of 8 and 2 of 8 mice in the 4 mg/kg and 10 mg/kg respectively (Figure 5).

### Selinexor treatment amplifies anoikis in prostatic cancer cells with high metastatic potential

**Figure 6:**
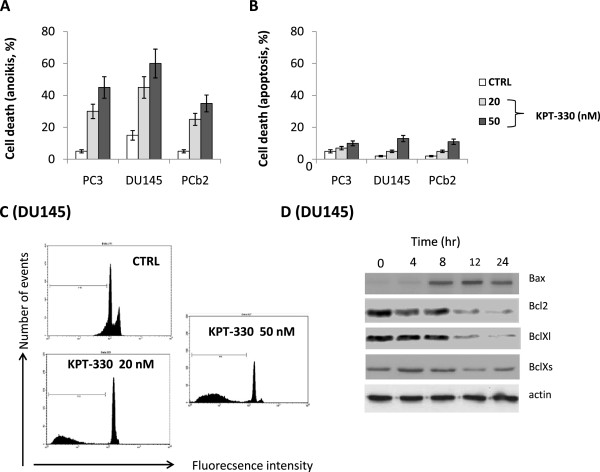
**In vitro experiments: Selinexor (KPT-330) amplified anoikis in prostatic cancer cells with high metastatic potential. (A)** this panel shows the cell death percentage in PC3, PCb2 and DU145 cells treated or not with 20 or 50 nM KPT-330, corresponding to IC10 and IC20 values for this compound [18], and grown in non adhesion conditions (anoikis). **(B)** This panel shows the cell death percentage obtained in the same cells treated or not with KPT and grown in adhesion conditions (apoptosis). **(C)** This panel show the FACS analyses evaluated in DU145 treated with 20 and 50 nM KPT-330 grown in non adhesion conditions (anoikis). **(D)** Selinexor reduces migration and tumor invasion by reducing MMP-9, MMP-2 and uPA secretion.

In order mimic the effects that Selinexor may have on cell viability of tumor cells which extravasate in the blood and lymphatic stream, we grew PC3, PCb2 and DU145 cells in polyhema-treated dishes which prevents cell attachment. We hypothesize that Selinexor may accelerate this apoptotic process determining a strong reduction of cells which can establish metastatic lesions. The lack of anchorage-dependent cell growth is associated to metastatic process both in the detachment and invasive (extravasation) phase. Anoikis is a form of programmed cell death which is induced by cells detaching from the surrounding extracellular matrix (ECM). Usually cells stay close to the tissue to which they belong since the communication between proximal cells as well as between cells and ECM provide essential signals for growth or survival. When cells are detached from the ECM, i.e. there is a loss of normal cell–matrix interactions, they may undergo anoikis. To verify if KPT330 was active inducing or amplifying anoikis, PCa cells were treated with sub-toxic concentrations of Selinexor (20 and 50 nM) corresponding to the IC10 and IC20 values for this compound [45]. We chose the dose of 50 nM since this is the pharmacological plasma concentration obtained administrating 4 mg/Kg day. In Figure 6A, we show that the percentage of cells which died following Selinexor treatment in presence of polyhema was 3.6 fold higher compared to cells grown in adhesion conditions (Figure 6B), suggesting that a portion of “extravasate cells” could die during treatment with Selinexor. In Figure 6C we show the anoikis FACS profile collected from DU145 and treated with 20 and 50 nM Selinexor. Anoikis was associated with modulation of bcl2 family members as indicated in Figure 6D. In particular, Bcl2 and BclXl levels were significantly reduced in the time by Selinexor treatment whereas BclXs and Bax levels were increased.

### Selinexor reduces migration and tumor invasion by reducing MMP-9, MMP-2 and uPA secretion

**Figure 7:**
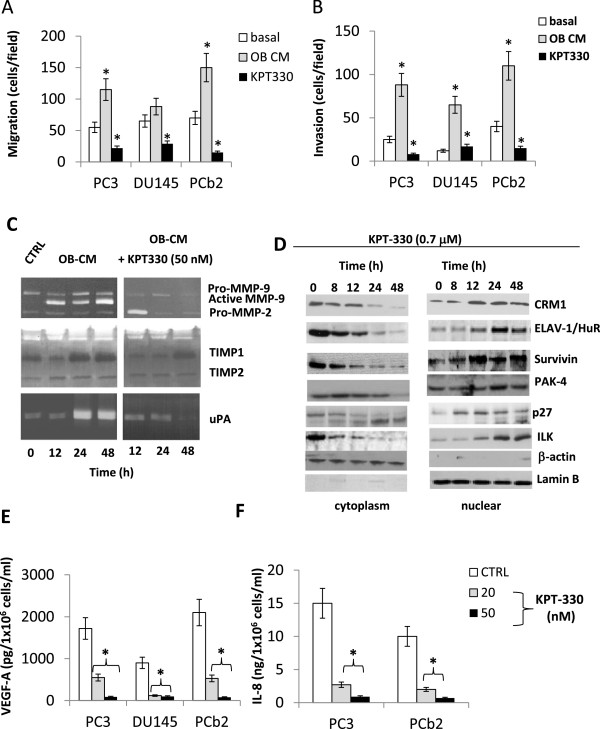
**Selinexor (KPT-330) reduces migration and invasion of DU145, PCb2 and PC3 tumor cells. (A)** migration test with treatment with 50 nM KPT-330 by using Boyden chambers and osteoblast conditioned medium as chemotractant. **(B)** invasion test in the same experimental conditions. **(C)** Deregulation of MMP-9, MMP-2 and uPA secretion after selinexor treatment. **(D)** time-dependent variation in the levels nuclear and cytoplasm levels of P27 and ILK after treatment with selinexor. Each Western Blot lane was loaded with 40 μg of proteins and normalized vs b-actin (cytoplasm levels) and lamin B (nuclear levels). **(E)** VEGF measured by ELISA and performed on conditioned media from cells treated or not with 20 or 50 nM selinexor. **(F)** Il-8 measured by ELISA and performed on conditioned media from cells treated or not with 20 or 50 nM selinexor.

To study the effects of Selinexor on tumor cell migration and invasion, specific assays by using Boyden chambers were performed. Cells were pre-treated with Selinexor and in the inferior compartment of Boyden chamber osteoblast conditioned medium was used as chemotractant. Also in this case we chose the dose of 50 nM since this is the pharmacological plasma concentration obtained administrating 4 mg/Kg day We demonstrated that osteoblast conditioned medium increased significantly cell migration and invasion of PC3, DU145 and PCb2 cells and Selinexor (50 nM) efficiently inhibited cell migration and matrigel invasion (Figure 7A,B). Interestingly, reduced migratory and invasive properties were coupled with down regulation of MMP-9, MMP-2 and uPA secretion (Figure 7C), with reduced protease/tissue inhibitor ratios. Immuno-cytochemical and western blot analyses performed on cell extracts from cultured cells with or without Selinexor (50 nM), revealed an increased and prevalent nuclear localization of p27, survivin, CRM1, ELAV-1, ILK and PAK-4, whereas controls showed a low level and cytoplasmic-prevalent localization (Figure 7D). Survivin has been implicated in cell division and apoptosis. Its cytoplasmic localization is essential for their inhibitory effects on caspases activity and apoptosis. CRM1 activity and localization is modulated by Akt/mTOR pathways. Increased nuclear localization of CRM1 by TORC1/TORC2 [46] or pan HDAC [47] inhibitors has been associated to apoptotic effects of these compounds through reduced survivin levels in the cytoplasm. Interestingly, Selinexor also increased the nuclear expression of ILK and PAK-4, two proteins associated with the migration process, with a time-dependent decrease in the expression levels of total ILK (Figure 7D). We analyzed p27 and ILK expression in cytoplasm and nucleus extracts by using a protein loading control β-actin and lamin B, respectively. The same loading controls were used to verify the purity of cytoplasm and nucleus extracts.

### Selinexor reduces tumor angiogenesis and osteoclast differentiation and activity in RAW264.7

**Figure 8:**
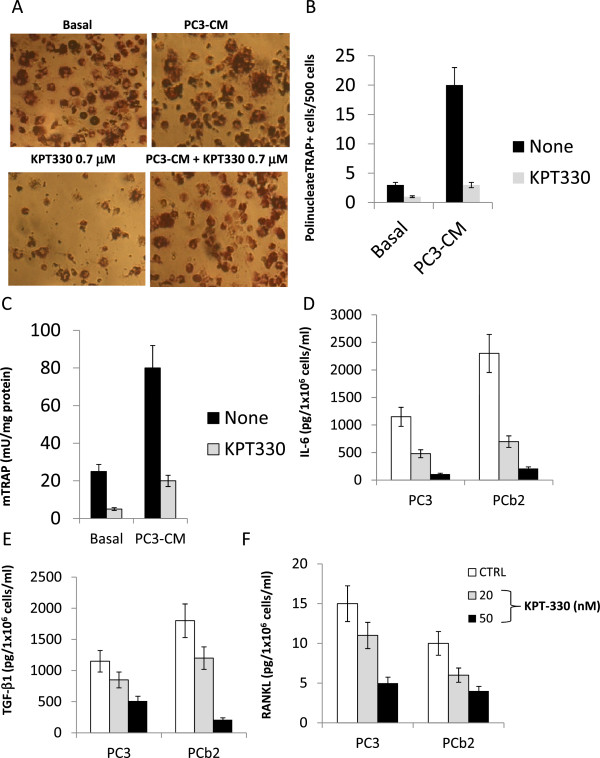
**Selinexor (KPT-330) inhibits PCa cells-Induced osteoclast differentiation in the RAW264.7 cell model. (A)** cytochemical evaluation of mouse TRAP in RAW264.7 cells treated with PC3 conditioned medium when these cells were cultured in the presence or not of 0.7 μM selinexor. This concentration was the IC20 value for RAW264.7 cell and was easily reached in vivo when 10 mg/Kg (q 2d × 3 weeks) KPT-330 was administered in the mice. Osteoclasts are large polinucleated TRAP + cells. **(B)** TRAP + polinucleated cells were counted and plotted in a graph considering a total count of 500 cells. **(C)** cells were collected and lysed to evaluate the enzymatic mTRAP activity. **(D)** IL-6, TGFb1 **(E)** and **(F)** RANL levels measured in osteotropic PC3 and PCb2 cells treated with 20 and 50 nM selinexor.

To examine the effect of Selinexor on tumor angiogenesis, the expression levels of angiogenetic cytokines (IL-8 and VEGF) were measured in conditioned medium (CM) collected from PC3 and DU145 cultured with SDF-1α cells and treated with Selinexor. Significantly higher levels of IL-8 and VEGF were found in controls compared with Selinexor treated cells (Figure 7E,F). PCa cell-induced osteoclastogenesis was analyzed on monocyte-like RAW264.7 cells by using conditioned medium (CM) collected from PC3 cells untreated or treated with CTRL or with Selinexor. RANKL, at the concentration of 5 ng/ml, was used to boost the osteoclastogenesis and 5 days later, the levels of mouse tartrate-resistant acid phosphatase (mTRAP), a marker of osteoclast differentiation, was measured in RAW264. 7 cells by enzymatic assay performed in total extracts. High levels of mTRAP were found in cultures treated with CM from untreated PC3 cells while lower levels were found in RAW264.7 cells treated with CM from Selinexor treated PC3 cells (Figure 8A-C). Negligible, if any, differentiated osteoclasts were observed in RAW264.7 cells treated with 5 ng/ml RANKL alone. Selinexor showed antiproliferative effects on RAW264.7 (data not shown) In the context of bone metastasis, however, tumor cells could induce the expression of osteoclastogenic factors [42, 48, 49]. Therefore, we tested whether selinexor could modulate the ability of PC3 to secrete osteotropic cytokines such as IL-6, IL-8, TGF-β1 and RANKL. We showed that PC3 cells treated with Selinexor, at a low concentrations (IC20), produced significantly lower IL6 (Figure 8D) with reduced effects on the expression of other pro-osteoclastogenic cytokines, including RANKL [46], IL-8 [47], TGF-β1 [48] (Figure 8E-G).

## Discussion

PCa cells are highly dependent upon the microenvironment where cytokines and contact with stromal cells promote cell activation and proliferation as well as resistance to spontaneous and drug-mediated apoptosis. Like many oncogenic and growth related pathways, many of these microenvironment-activated pathways intersect with TSPs which are exported from the nucleus by XPO1, leading to their functional inactivation [7, 11–16]. XPO1 inhibition leads to CRM1 nuclear localization with forced accumulation of TSPs in the nucleus determining reduced oncogenic functions. Thus, the concept of inhibiting XPO1 has been explored as a potential therapeutic intervention using clinically relevant orally bioavailable SINE compounds. We have previously demonstrated that the inhibition of XPO1 with SINE reduced the cell and tumor growth in PCa preclinical models inducing G2/M cell cycle accumulation and apoptosis [45]. In this report we used: (i) DU145 cell line, injected orthotopically in prostate gland of SCID mice, to study the anti-metastatic effects of KPT330 and KPT251; (ii) PCb2 cell lines, derived from PC3 cells after serial intra-bone growth in mice, intra-ventricularly injected in male nude mice to mimic an aggressive prostate cancer with no evident bone metastasis and (iii) luciferase transfected PC3 cells for the intratibial injection to study the intra-bone tumor growth. The choice to use two different PC3 derivatives arose by the experimental convenience to obtain the higher incidence rate of bone lesions with PCb2 cells, as widely demonstrated by our research group [29, 30] when the intracardiac model was used and by the fact that luciferase transfected PC3-M-pro4 cells were widely characterized [28] and kindly provided from G van der Pluijg. The use of luciferase transfected cells serve to identify little not radiologically evident bone lesions also if bioluminescence analysis has the main disadvantage to have a low spatial resolution whereas bioluminescence intensity is proportional to cells present in a tumor. Therefore bioluminescence allows to have a good quantitation of tumor burden when PC3 cell growth destroys the bone and extents outside of it. X-ray is a well-established and referenced modality to longitudinal monitor bone lesions in small animals with a good spatial resolution. Intracardiac experiments showed very few fractures and extra-bone lesion before animals died whereas intratibial experiments showed larger bone lesions with fractures and extra-bone growth. For this reason the non- use of PC3-luc cells in the intra-ventricular injection has allowed us to have an easy and direct comparability with those obtained in humans. Inter- and intra-observer variability have been partially mitigated by the use of Image-J, a software widely used in the objective quantification of bone lesion extension.

To our knowledge, this manuscript is the first report showing that SINE XPO1-antagonists show anti-metastatic properties. Our data suggest that SINE compounds, and Selinexor in particular, could achieve these effects through several mechanisms: (i) inhibiting the survival and inducing apoptosis of circulating metastasizing cells; (ii) reducing the migration and the invasion of metastasizing cells; (iii) reducing bone colonization by metastatic cells; (iv) reducing the tumor burden both in the primary and in bone sites; (v) reducing the survival and probably differentiation of osteoclast progenitors. The first two statements born from data shown in Figures 6 and 7 in which we observed that Selinexor, a clinically relevant potent and oral available XPO1 inhibitor, was able to increase apoptosis (anoikis) when PC-b2 and DU145 tumor cells were seeded in tissue culture dishes coated with poly HEMA and thus forced to grow in suspension. Under these conditions, indeed, both the number and the size of cell spheroids were significantly reduced and apoptosis was significantly greater after Selinexor treatment when compared with controls. Selinexor was also able to inhibit PC3, PCb2 and DU145 cell migration, and this correlated with p27 and ILK nuclear accumulation in agreement with data reported by Wang [50] and Nakrieko [51], respectively demonstrating that the cell cycle inhibitor p21-Cip and p27-Kip proteins play unique roles outside of the nucleus regulating actin dynamics and cell migration. In addition we observed also a significant decrease of cytoplasmic PAK4, a well known kinase involved in β-catenin mediated Epithelial Mesenchymal Transition (EMT), after selinexor treatment [52]. These results are strongly suggestive for a reduced invasive capacity of selinexor treated cells. Moreover, selinexor treatment induced segregation of CRM1 and ELAV1/HuR in the nucleus. Being ELAV1/Hur a member of RNA-binding proteins that selectively bind AU-rich elements, AREs, found in the 3′ untranslated regions of mRNAs, the loss of cytoplasmatic shuttling could destabilize ARE-containing mRNAs [53] and reduced protein synthesis including angiogenetic cytokines, VEGF-a and IL8, and proteases involved in the degradation of extracellular matrix proteins such as MMP-9, MMP-2 and uPA. Effectively, also in PCa cells we demonstrated that selinexor inhibited VEGF-a and IL-8 secretion as well as the expression and activity of MMP-9, MMP-2 and uPA as has been demonstrated for the natural product XPO1 inhibitor leptomycin B [54, 55]. However, the current report is the sole evidence that clinically relevant, small molecule SINE compounds reduce MMP-9 and angiogenesis in PCa. ELAV1 has been, indeed, indicated as a potentially useful target for cancer diagnosis, prognosis, and therapy of tumors [56].

In vivo experiments demonstrated that Selinexor and KPT-251 reduced also tumor growth and visceral metastases when DU145 cells were injected in the prostate gland (orthotopic model) of mice. The reduced incidence of visceral metastases was also associated to reduced angiogenesis evaluated as vessel count performed in prostate glands and seminal vesicles containing DU145 tumors and this was in agreement with our in vitro results. This anti-angiogenic effect provides a further obstacle to extravasation/intravasation of metastatic tumor cells. We demonstrate also that visceral metastases and osteolytic lesions were also reduced after treatment with selinexor or KPT251 when the intra-ventricular model were used. Selinexor resulted to be more potent of KPT251. This determined a significant increase in the disease-free survival and overall survival of treated mice. Similarly, selinexor and KPT251 affected intra-osseous tumor growth when PC3 cells were inoculated directly in the tibia where these agents impaired also osteoclastogenesis. In this model, areas of osteolytic lesions and tumor growth were significantly reduced by CRM1 inhibitors: also in this case KPT330 resulted to be more potent respect to KPT251. The degree of bone resorption in bone metastases influences the course of disease and serum markers of N-telopeptide type I collagen (CTX) has been shown to be predictive of SREs, bone disease progression, and death [57]. Therefore we analyzed CTX-I and mTRAP, a marker of osteoclasts, to have a further indication of reduction of tumor-derived bone osteolysis. We found that Selinexor and KPT-251 significantly reduced the levels of CTX-I in PCa bearing mice. In addition, also mTRAP levels were significantly reduced by XPO1 inhibition. These data are in agreement with Tai and coworkers [58] demonstrating that XPO1 inhibition induced tumor cell cytotoxicity and impaired osteoclastogenesis in multiple myeloma. These authors found that XPO1 SINEs directly impaired osteoclastogenesis and bone resorption with minimal impact on osteoblasts and bone marrow stromal cells. Extending the data reported by Tai and coworkers, we observed that XPO1 inhibition can mediate a reduction in osteolysis directly through effects on bone cells. We observed that count of TRAP positive cells (osteoclasts) in the bone metastases were significantly reduced after treatments as reduced was the growth of osteoclast precursors, modeled by using monocyte-macrophage RAW 264.7 cells. RANKL induced TRAP activity and the formation of multinucleated cells (osteoclasts) in RAW 264.7 cell population and this phenomenon was significantly reduced by Selinexor administration suggesting that osteclasts was not produced. We observed also that Selinexor induced cytotoxic effects on RAW264.7 cells that were lower, in terms of inhibition percentage, to those observed for osteoclast formation suggesting that selinexor could affect also osteoclast diffentiation in agreement with Tai and coworkers [58]. Furthermore, we observed that selinexor reduced also RANKL secretion by tumor cells in agreement with the report by Tai and coworkers in myeloma [58]. However, this suggestions are not conclusive to state that selinexor shows main effects on osteoclasts to determine reduction of osteolysis. Progression of the established skeletal PCa tumors is, indeed, supported by the mutual interplay between locally increased osteoclastic [11, 16] and osteoblastic [36, 59] activity and tumor cell proliferation (the “vicious cycle;”), which may also facilitate earlier steps in skeletal tumor establishment and development. Therefore, SINE induced reduction in tumor progression observed following RANKL inhibition is likely the consequence of interrupting the “vicious cycle,” which leads to a reduction in local concentrations of bone-derived factors as a result of lower osteoclastic bone resorption. As osteoclast-mediated bone destruction has been reduced by SINE treatment, the reactive osteoblastic activity is also reduced. That report in myeloma is the sole report, in addition to ours, demonstrating that SINEs impair bone remodeling after primary cancer (myeloma) and bone metastasis (prostate cancer). Taken together, these effects translate in the reduced incidence of metastases and SREs and increased overall survival of SINE-treated animals.

## Conclusions

Our data show that selective blockade of XPO1-dependent nuclear export with small molecule, drug-like SINEs represents a completely novel approach for the treatment of subjects with primary tumors at high risk of visceral and bone metastatic spread. The SINE compounds actively prevent visceral and bone metastasis of PCa cells, and impair PCa induced osteoclastogenesis and angiogenesis, suggesting they could be relevant in treating metastatic PCa including patients with SREs. Further studies should be addressed on the effects of selinexor on bone calcification in controls (tumor-free) mice in the short and long term observation. This could help to define the role of XPO1 on the osteoclastogenesis and of SINEs on prevention of osteoclastogenesis in normal bone, but this is not the topic of the present report. Selinexor, given orally, is now in Phase 1 clinical trials in patients with advanced solid tumors including patients with advanced PCa (clinicaltrailas.gov:NCT01607905).
